# Body composition and nutritional and metabolic parameters in postmenopausal women sufficient, insufficient and deficient in vitamin D

**DOI:** 10.20945/2359-3997000000121

**Published:** 2019-04-15

**Authors:** Luisa Amábile Wolpe Simas, Leila Caroline Bianchet Zanatta, Carolina Aguiar Moreira, Victoria Zeghbi Cochenski Borba, Cesar Luiz Boguszewski

**Affiliations:** 1 Universidade Federal do Paraná Serviço de Endocrinologia e Metabologia Departamento de Medicina Interna Universidade Federal do Paraná Curitiba PR Brasil Serviço de Endocrinologia e Metabologia (SEMPR), Departamento de Medicina Interna , Universidade Federal do Paraná , Curitiba , PR , Brasil

**Keywords:** Vitamin D, body composition, nutrition, metabolic syndrome, menopause

## Abstract

**Objective:**

We investigated changes in body composition and nutritional and metabolic parameters in a group of postmenopausal women who were classified as sufficient, insufficient and deficient in vitamin D.

**Subjects and methods:**

A total of 106 postmenopausal women were included in this cross-sectional study and classified according to their serum levels of 25-OH-vitamin D as sufficient (≥ 30 ng/mL; group S), insufficient (20.1 and 29.9 ng/mL; group I) or deficient (≤ 20 ng/mL; group D) in vitamin D. Body composition was measured by dual-energy X-ray absorptiometry (DXA); dietary recall questionnaires were completed; and blood samples were analysed to compare the metabolic and nutritional status of the study groups.

**Results:**

Eleven (10.4%) of the women were classified in group S, 50 (47.2%) in group I and 45 (42.4%) in group D, with a mean serum level for 25-OH-D of 21.1 ± 7.0 ng/mL in all participants. Body composition did not differ among the groups. Serum levels of 25-OH-D were negatively correlated with serum levels of triglycerides, total cholesterol and LDL cholesterol.

**Conclusions:**

Vitamin D insufficiency and deficiency were highly prevalent in our group of postmenopausal women, showing an association with an unfavourable lipid profile.

## INTRODUCTION

Vitamin D deficiency is a worldwide public health problem (1), even in regions and areas with sun exposure for most of the year. In Brazil, several studies have confirmed a high prevalence of hypovitaminosis D (2,3). Serum vitamin D levels may be affected by a number of factors (4), and there is a higher prevalence of vitamin D deficiency in women, with the highest number of daily cases in countries with higher latitude and in people with increased pigmentation of the skin (5). Vitamin D deficiency has been associated with several metabolic and cardiovascular abnormalities, including hypertension, diabetes mellitus, metabolic syndrome, obesity, vascular inflammation, left ventricular hypertrophy and congestive heart failure (6-8). During menopause, women may be particularly susceptible to the consequences of vitamin D deficiency since in this period of life oestrogen decreases, resulting in decreased bone mineral density (BMD) and lean mass, increased fat mass, and an increased risk of metabolic syndrome (MS) and cardiovascular morbidities (9-12). According to the International Diabetes Federation (IDF), the diagnosis of MS requires the presence of abdominal obesity associated with two of the following criteria: arterial hypertension, hypertriglyceridemia, reduced HDL-cholesterol, and elevated fasting glycaemia (13). The abdominal obesity associated with MS is a well-established cardiovascular risk factor and is frequently associated with hypovitaminosis D (12).

Nevertheless, there have been divergences among the studies on the relationship between serum vitamin D levels and the various components of MS and body composition (14-16). Thus, the objective of this cross-sectional study was to compare the body composition, nutritional factors and metabolic parameters related to MS in menopausal women sufficient, insufficient and deficient in vitamin D.

## SUBJECTS AND METHODS

A cross-sectional descriptive study was carried out with postmenopausal women who were recruited from outpatient clinics of the Endocrine Division (SEMPR) of the Federal University Hospital in Curitiba, South of Brazil, between February 2012 and March 2013. Menopause was defined through the 12-month criterion of amenorrhea (absence of menstruation) (17). The exclusion criteria were women in the menacme; those older than 70 years; patients with known diabetes mellitus, renal, hepatic or cardiorespiratory dysfunctions, bone diseases, secondary osteoporosis or neoplasias; and women in use of any medication in the last 6 months that according to medical judgement could interfere with glycaemia and/or bone metabolism.

The study was approved by the Human Research Ethics Committee of our academic institution. All participants were informed about the research procedures and signed the informed consent term.

### Clinical evaluation

The clinical evaluation included the collection of personal data, general clinical status with emphasis on concomitant medications, presence of chronic diseases, blood pressure (BP), waist circumference (WC), weight, height and body mass index (BMI). The number of calories ingested daily through food was calculated using data from the 24-hour food recall and Clinical Diet Win ^®^ Software 2002 (15). The measurements were performed according to WHO recommendations (18) using a 150 kg scale with an accuracy of 1 kg and a stadiometer with an accuracy of 1 mm. The BP was measured twice by the auscultatory method, with the participants seated, and it was defined as normal when less than 130/85 mmHg and abnormal when equal or greater than this cut-off (18). The study group was classified according to their BMI in: normal weight 18.0-24.9 kg/m ^2^ ; overweight 25.0-29.9 kg/m ^2^ ; obese grade 1 30.0-34.9 kg/m ^2^ , obese grade 2 35.0-39.9 kg/m ^2^ and obese grade 3 ≥ 40 kg/m ^2^ (19). WC was considered normal if less than 80 cm and altered if equal or greater than 80 cm. A WC between 80 and 87 cm was considered of an increased risk for cardiovascular events in overweight/obese women and equal or above 88 cm was considered of very high increased risk (20,21).

### Laboratory tests

Peripheral venous blood samples were collected after 8 hours of fasting and stored at -80 °C for analysis. Blood glucose was evaluated by hexoquinase/G-6-PD and values ≥ 100 mg/dL were considered abnormal; serum insulin levels were determined by chemiluminescence (CL), with values of reference (VR) of 25 to 30 uUI/mL and intra-assay coefficient of variation (CV) < 10%. HOMA-IR was calculated with the following formula: glycemia (mg/dL) X insulin (uUI/mL)/405. C-reactive protein (CRP) was measured by the turbidometry method, with a limit of detection below 0.5 mg/dL and CV < 10%. The colorimetric enzymatic method with a VR < 200 mg/dL was used for the determination of total cholesterol. HDL-cholesterol was measured by direct/homogeneous method and values ≤ 50 mg/dL were considered abnormal. LDL-cholesterol was determined by the Friedwald formula and scored at optimal values when ≤ 100 mg/dL, desirable at values between 101-129 mg/dL, borderline 130-159 mg/dL and high ≥ 160 mg/dL. Serum triglycerides (TG) was measured by the glycerol-phosphate oxidase method and hypertriglyceridemia was defined by levels ≥ 150 mg/dL. Creatinine was evaluated by the picratecalcalino method with a VR of 0.57-1.11 mg/dL. TGO and TGP were evaluated by NADPH, with a VR varying between 0-55 IU/L and 5-34 IU/L, respectively.

Serum calcium was determined by the Arsenazzo III method with a VR of 8.6-10.3 mg/dL and serum phosphorus by the UV phosphomolybdate method with a VR of 2.3-4.7 mg/dL. All measurements of PTH and vitamin D [25(OH)D] were determined simultaneously in one run by CL, with a VR of 11.7-61.1 pg/mL and CV < 10% for PTH and VR of 30-100 ng/mL and CV < 10% for 25(OH)D. According to the 25(OH)D measurements, the participants were classified as having vitamin D deficiency when serum levels were ≤ 20 ng/ml, insufficiency when levels were between 20.1 and 29.9 ng/mL and sufficiency when ≥ 30 ng/mL (22).

### Body composition

Measurements of lumbar spine (L1 to L4) and total femoral BMD, as well as those of body composition (percentage total fat), were performed with a properly calibrated Lunar Prodigy Advance (GE Healthcare, Madison, WI, USA), with a coefficient of variation of 2% and by the same examiner.

### Statistical analysis

Statistical analyses were performed with the statistical package GraphPad Prism. All data are presented as the mean ± SD, except as otherwise noted. To analyse the normality of the data, the Shapiro-Wilk test was used, which resulted in non-normal data. Thus, non-parametric tests were used to analyse the variables.

For comparative analysis between groups, the Friedman test (with Dunn’s a posteriori test) was used for the quantitative variables. In the case of qualitative variables, the chi-square test was used. In the insufficient and deficient groups, the Mann-Whitney test was used for comparisons of the quantitative variables, and the chi-square test and Fisher’s exact test were used for the qualitative variables.

For the tests between variables, Pearson’s correlations were used, following the theorem of large numbers. From the correlation index, shape and direction can be interpreted according to the concept that directly proportional correlations have a positive sign since inversely proportional correlations have a negative sign (23). In all cases, a level of significance of 5% (p < 0.05) was considered.

## RESULTS

The study group consisted of 106 postmenopausal women with a mean (±SD) age of 56.5 ± 6.1 years (range 43-70) and BMI of 28.2 ± 4.9 kg/m ^2^ (range 19.7-41.1). In the whole group, serum levels of 25(OH)D were 21.1 ± 7.0 ng/mL (range 7.5-55.0), with 11 women (10.4%) classified as sufficient, 50 (47.2%) as insufficient and 45 (42.4%) as deficient in vitamin D. The main clinical features of the three study groups are summarized in Table 1. There were no significant differences among the groups.

**Table 1:** Clinical characteristics of postmenopausal women classified as sufficient, insufficient and deficient in vitamin D

Characteristic		Sufficient (n = 11)	Insufficient (n = 50)	Deficient (n = 45)*
Age (yrs)		54.9 ± 5.9	56.2 ± 5.4	57.3 ± 6.9
Body mass index (BMI, kg/m ^2^ )		28.7 ± 5.4	28.9 ± 4.9	27.4 ± 4.7
BMI classification (%)				
Normal		27.3	18	33.3
Overweight		36.4	46	37.8
Obesity grade I		18.2	22	20
Obesity grade II		18.2	12	8.9
Obesity grade III		0	2	0
Waist circumference (cm)		91.2 ± 13.0	93.8 ± 12.4	89.5 ± 9.2
Cardiovascular risk (%)				
Normal (WC ≤ 80 cm)		27.3	10	20
High (WC 81-87 cm)		9.1	18.0	24.4
Very high (WC ≥ 88 cm)		63.6	72.0	55.6
Blood pressure (%)				
Abnormal		54.5	44	35.6
Normal		45.5	56	64.4

* All p values were > 0.05.

Table 2 shows the comparisons between anthropometric measurements and body composition parameters evaluated by DXA in the three study groups. Again, no statistically significant differences were identified among vitamin D sufficient, insufficient and deficient postmenopausal women. The results of the laboratory tests (Table 3) showed a difference only in the insufficient and deficient groups in relation to serum calcium levels and TGP. Serum calcium was significantly higher in the deficient group (9.4 ± 0.5 vs. 9.2 ± 0.3 mg/dL in the insufficient group, p = 0.04), whereas serum TGP was significantly lower in the deficient group (18.4 ± 9.6 vs. 24.0 ± 16.2 mg/dL in the insufficient group, p = 0.047).

**Table 2:** Anthropometric characteristics and body composition parameters by DXA assessment of postmenopausal women classified as sufficient, insufficient and deficient in vitamin D

Variable	Sufficient (n = 11)	Insufficient (n = 50)	Deficient (n = 45)
Gynoid adipose mass (%)	48.3 ± 5.9	48.5 ± 5.5	47.2 ± 6.5
Android adipose mass (%)	47.6 ± 10.9	48.2 ± 7.5	46.9 ± 8.0
Total adipose, %	41.9 ± 7.4	41.8 ± 6.0	40.5 ± 7.0
Truncal fat (%)	43.3 ± 8.6	43.8 ± 6.8	42.3 ± 7.3
BMD L1-L4 (g/cm ^2^ )	1.01 ± 0.10	1.07 ± 0.15	1.06 ± 0.13
BMD total femur (g/cm ^2^ )	0.90 ± 0.10	0.96 ± 0.15	0.92 ± 0.12
BMC L1-L4 (g)	50.8 ± 7.1	54.2 ± 10.7	53.6 ± 9.1
Bone mineral density (%)			
Normal	9.1	30.0	24.4
Osteopenia	81.8	50.0	51.1
Osteoporosis	9.1	20.0	24.4

BMD L1-L4: bone mineral density lumbar spine; BMC: bone mineral content.

* All p values were > 0.05.

**Table 3:** Characteristics of the biochemical exams of postmenopausal women classified as sufficient, insufficient and deficient in vitamin D

Variable	Sufficient (n = 11)	Insufficient (n = 50)	Deficient (n = 45)
Total cholesterol (mg/dL)	223.5 ± 37.2 (233.0)	209.3 ± 41.6 (208.0)	226.4 ± 46.5 (228.0)
Triglycerides (mg/dL)	113.7 ± 42.7 (92.0)	122.6 ± 56.9 (107.0)	135.6 ± 61.0 (124.0)
HDL (mg/dL)	52.1 ± 10.0 (52.0)	45.7 ± 10.2 (44.0)	47.9 ± 12.5 (47.0)
LDL (mg/dL)	148.8 ± 34.2 (154.0)	139.8 ± 38.0 (139.5)	151.3 ± 39.4 (152.0)
Glycemia (mg/dL)	95.0 ± 9.6 (92.0)	93.9 ± 12.2 (92.0)	90.7 ± 8.7 (89.0)
Insulin (uUI/mL)	10.4 ± 5.3 (11.1)	10.9 ± 6.1 (9.4)	10.1 ± 5.9 (7.8)
HOMA-IR	2.47 ± 1.30 (2.36)	2.64 ± 1.78 (2.15)	2.33 ± 1.58 (1.85)
Creatinine (mg/dL)	0.73 ± 0.08 (0.7)	0.73 ± 0.12 (0.7)	0.71 ± 0.10 (0.7)
TGO (UI/L)	20.2 ± 7.3 (18)	20.8 ± 9.5 (18)	19.0 ± 5.5 (18)
TGP (UI/L)	20.1 ± 6.8 (20)**	24.0 ± 16.2 (19)	18.4 ± 9.6 (16)
Albumin (mg/dL)	4.07 ± 0.20 (4.0)	3.96 ± 0.22 (4.0)	3.96 ± 0.21 (4.0)
Calcium (mg/dL)	9.35 ± 0.27 (9.4)	9.20 ± 0.34 (9.2)	9.38 ± 0.50 (9.3)*
Phosphorus (mg/dL)	3.51 ± 0.45 (3.6)	3.43 ± 0.46 (3.3)	3.31 ± 0.62 (3.4)
PTH (pg/mL)	50.2 ± 11.5 (50.1)	56.3 ± 22.4 (50.8)	59.3 ± 17.7 (3.6)
ng/mL (mg/dL)	0.38 ± 0.10 (0.33)	0.45 ± 0.25 (0.33)	0.62 ± 1.05 (0.33)

HDL: high density lipoprotein; LDL: low density lipoprotein; HOMA-IR: homeostatic model assessment for insulin resistance; TGO: glutamic-oxalacetic transaminase; TPG: pyruvic glutamic transaminase; PCR-C: reactive protein; mg/dL: milligrams per decilitre; uUI/mL: international micro unit per millilitre; UI/L: intentional unit per litre; pg/mL: picogram per millilitre. Values are means ± standard deviations, with medians in parentheses or percentage frequencies. * p = 0.04 vs. insufficient. ** p = 0.04 vs. insufficient.

There were statistically significant inverse correlations between serum 25(OH)D levels with serum levels of TG (r = 0,27; p = 0,006), total cholesterol (r = -0,25; p = 0,011) and LDL (r = -0,22; p = 0,028).

The calcium dosage presented a significant difference when the insufficient and deficient vitamin D groups were compared. Thus, a correlation was performed between the three groups with the glycaemia values and the HOMA calculation to verify if the serum calcium value would be related to the other groups variables involved with insulin resistance and type 2 diabetes.

There were no significant differences between the three groups in relation to the SM criteria for the three groups nor when there was a comparison between the insufficient and vitamin D-deficient groups.

Through analysis of the 24-hour recall, it was possible to verify that the average dietary intake of vitamin D, calcium and cholesterol was lower in the insufficient group (Table 4).

**Table 4:** Dietary ingestion in postmenopausal women classified as sufficient, insufficient and deficient in vitamin D

24-hour recall	Sufficient (n = 11)	Insufficient (n = 50)	Deficient (n = 45)	P value
Vitamin D, µg	3.93 ± 0.80 (3.79)	0.84 ± 1.12 (0.43)	1.32 ± 0.90 (1.27)**	< 0.001*
Calcium, mg	395.0 ± 199.2 (379.3)	238.7 ± 174.1 (199.6)	248.6 ± 130.5 (234.3)	0.021*
Kcal/total, kcal	1649 ± 601.4 (1647)	1406 ± 608.1 (1257)	1985 ± 637.8 (2123)***	< 0.001*
Cholesterol, mg	314.2 ± 601.4 (207.5)	126.3 ± 94.3 (109.5)	199.9 ± 135.1 (168.1) ****	< 0.001*
Fibre (g)	8.6 ± 3.2 (8.0)	9.1 ± 5.5 (8.5)	7.7 ± 2.6 (7.7)	0.633

* p < 0.05. ** p = 0.026 vs. insufficient. *** p = < 0.001 vs. insufficient. **** p = 0.003 vs. insufficient.

## DISCUSSION

There was a high prevalence of hypovitaminosis D in the studied group, where only 10% of the 106 women had adequate vitamin D levels (considering a cut-off point above 30 ng/mL) and 90% were deficient or insufficient. These values are higher than those found in cohort studies of menopausal women, which showed rates of 67% and 68% for hypovitaminosis D (24,25). In another study with 93 postmenopausal women living in the city of Recife, the authors found a prevalence of 24% of vitamin D deficiency, which was lower than this study; this study also found a mean serum vitamin D concentration of 28.8 ng/mL, higher than the 21.1 ng/mL observed in our patients (with cut-off point for deficiency less than 20 ng/mL), because according to with the authors themselves the incidence of solar radiation is higher because Reef is closer to the equator (26).

The current study found an inverse correlation between serum vitamin D levels and serum levels of TG, total cholesterol and LDL-cholesterol, indicating a trend towards an unfavourable lipid profile in menopausal women with lower levels of vitamin D. However, we did not find significant differences when we analysed and compared the groups deficient and insufficient in relation to body composition and metabolic variables. Similarly, these groups did not differ when analysed according to the SM criteria presented (27).

A study conducted by Iranian researchers has shown that the serum vitamin D concentration was lower in subjects with MS, similar to this study, compared to individuals without MS (27). To identify the relationship between daily intake of vitamin D, serum 25(OH)D levels and prevalence of MS, these researchers compared the plasma and nutritional variables of 128 healthy individuals and 122 individuals with a diagnosis of MS (27). The results showed that 98.4% of the patients in the MS group and 88.3% of the healthy individuals presented vitamin D deficiency [25(OH)D < 20 ng/mL]. This difference was statistically significant (p = 0.005). Furthermore, concentrations of 25(OH)D were significantly associated with waist circumference (p = 0.001), HDL (p = 0.001) and triglyceride levels (p = 0.012). The authors concluded that there is an association between vitamin D and MS. This study, however, failed to confirm the relationship between vitamin D deficiency and a higher prevalence of MS.

A study evaluating micronutrient concentrations in women (n = 8,137) who participated in the National Health and Nutrition Examination Survey III (NHANES III) showed that in postmenopausal women there was a negative association of vitamin D with BMI (25,28). Other researchers have observed that the percentage of body fat was also strongly associated with a low vitamin D concentration (23). The present study did not demonstrate a negative association with BMI and fat percentage.

Vitamin D deficiency has been associated with the development of cardiovascular diseases, hypertension, neoplasias, and diabetes, suggesting a crucial role for this vitamin in several homeostasis systems (29). Screening for vitamin D deficiency is cost-effective in some specific populations – postmenopausal, elderly, institutionalized, and pregnant women. In this study, vitamin D levels were not related to blood pressure and glycaemic indexes.

We observed differences between serum levels of Ca and TGP among the women who were insufficient and deficient in vitamin D; there were higher values of Ca and lower values of TGP. Ca intake was also significantly higher in the deficient group compared to the insufficient group, as was as the ingestion of cholesterol-containing foods and total daily caloric intake. It is known that adequate plasma concentrations of 25(OH)D are required to allow adequate absorption of calcium administered orally. A recent study showed that calcium absorption was 65% higher when plasma concentrations of 25(OH)D were in the mean of 34,8 ng/mL (30).

Although diet represents only 10% to 20% of the vitamin D necessary for the proper functioning of the organism, the dietary sources of vitamin D are scarce, and individuals depend mainly on the skin for production vitamin D, catalysed by solar UVB rays (31).

Vitamin D is found in food sources such as cod liver oil and fatty fish (salmon, tuna, and mackerel) or through endogenous cutaneous synthesis, which is the main source of this vitamin for most humans (31).

Vitamin D intake was higher in women whose levels were insufficient. Recent studies in the literature have correlated vitamin D supplementation with lower mortality rates; according to these data, we can suggest supplementation for correction of hypovitaminosis D and dietary inadequacy aimed at postmenopausal women (32,33).

In conclusion, in this group of menopausal women, there were no significant differences in body composition and metabolic parameters between women with insufficient and deficient levels of vitamin D. Further, lower vitamin D levels were associated with an unfavourable lipid profile; there was a high prevalence of hypovitaminosis D, corresponding to 90% of the sample; and serum vitamin D levels were influenced by dietary intake of vitamin D.
